# Supplementary material for: A Qualitative Study Exploring the Rehabilitation Experience of Individuals with a Previous Diagnosis of Cancer and/or Sepsis, Their Caregivers, and Health Providers
Source: Healthcare (Basel). 2025 Apr 4;13(7):822. doi: 10.3390/healthcare13070822 (PMC11988954; doi:10.3390/healthcare13070822)
Supplement: Supplementary file 1 [file healthcare-13-00822-s001.zip › healthcare-3524871-supplementary.pdf]

Supplementary File S1

COREQ (COnsolidated criteria for REporting Qualitative research) Checklist

A checklist of items that should be included in reports of qualitative research. You must report the page number in your manuscript where you consider each of the items listed in this checklist. If you have not included this information, either revise your manuscript accordingly before submitting or note N/A.

Topic	Item No.	Guide Questions/Description	Reported on Page No.
**Domain 1: Research team and reflexivity**			
Personal characteristics			
Interviewer/facilitator	1	Which author/s conducted the interview or focus group?	3-4
Credentials	2	What were the researcher's credentials? E.g. PhD, MD	3-4
Occupation	3	What was their occupation at the time of the study?	3-4
Gender	4	Was the researcher male or female?	3-4
Experience and training	5	What experience or training did the researcher have?	3-4
Relationship with participants			
Relationship established	6	Was a relationship established prior to study commencement?	NA
Participant knowledge of the interviewer	7	What did the participants know about the researcher? e.g. personal goals, reasons for doing the research	NA
Interviewer characteristics	8	What characteristics were reported about the inter viewer/facilitator? e.g. Bias, assumptions, reasons and interests in the research topic	NA
**Domain 2: Study design**			
Theoretical framework			
Methodological orientation and Theory	9	What methodological orientation was stated to underpin the study? e.g. grounded theory, discourse analysis, ethnography, phenomenology, content analysis	2
Participant selection			
Sampling	10	How were participants selected? e.g. purposive, convenience, consecutive, snowball	3
Method of approach	11	How were participants approached? e.g. face-to-face, telephone, mail, email	3
Sample size	12	How many participants were in the study?	4
Non-participation	13	How many people refused to participate or dropped out? Reasons?	4
Setting			
Setting of data collection	14	Where was the data collected? e.g. home, clinic, workplace	3-4
Presence of non-participants	15	Was anyone else present besides the participants and researchers?	3-4
Description of sample	16	What are the important characteristics of the sample? e.g. demographic data, date	4-5
Data collection			
Interview guide	17	Were questions, prompts, guides provided by the authors? Was it pilot tested?	Suppl 2
Repeat interviews	18	Were repeat inter views carried out? If yes, how many?	NA
Audio/visual recording	19	Did the research use audio or visual recording to collect the data?	3-4
Field notes	20	Were field notes made during and/or after the inter view or focus group?	3-4
Duration	21	What was the duration of the inter views or focus group?	3-4
Data saturation	22	Was data saturation discussed?	4
Transcripts returned	23	Were transcripts returned to participants for comment and/or	NA

Topic	Item No.	Guide Questions/Description	Reported on Page No.
		correction?	
**Domain 3: analysis and findings**			
Data analysis			
Number of data coders	24	How many data coders coded the data?	4
Description of the coding tree	25	Did authors provide a description of the coding tree?	4
Derivation of themes	26	Were themes identified in advance or derived from the data?	NA
Software	27	What software, if applicable, was used to manage the data?	4
Participant checking	28	Did participants provide feedback on the findings?	NA
Reporting			
Quotations presented	29	Were participant quotations presented to illustrate the themes/findings? Was each quotation identified? e.g. participant number	5-8
Data and findings consistent	30	Was there consistency between the data presented and the findings?	5-8
Clarity of major themes	31	Were major themes clearly presented in the findings?	5-8
Clarity of minor themes	32	Is there a description of diverse cases or discussion of minor themes?	5-8

Developed from: Tong A, Sainsbury P, Craig J. Consolidated criteria for reporting qualitative research (COREQ): a 32-item checklist for interviews and focus groups. *International Journal for Quality in Health Care*. 2007. Volume 19, Number 6: pp. 349 – 357

Once you have completed this checklist, please save a copy and upload it as part of your submission. DO NOT include this checklist as part of the main manuscript document. It must be uploaded as a separate file.

Supplementary File S2: Interview and Focus Group Scripts

Semi-Structured Interview Guide – Patient

(**note this interview guide was adapted for caregivers and health professionals)*

Thank you very much for agreeing to take part in this interview today. As described in the information and consent form, this interview consists of about 10 main questions and will take approximately 30-60 minutes. You do not have to answer any question you are not comfortable answering and we can stop the interview at any time.

1. To start off, can you please describe your experience with cancer and sepsis.

2. What are your current concerns regarding these conditions?

3. Frailty occurs when an individual has decreased function across multiple body systems. Individuals may notice reduced walking speed, weight loss, decreases in muscle mass, and decreased activity levels. Have you been told you have frailty or experienced any of these symptoms?
    a. If yes, when did this occur in relation to your cancer treatment and sepsis diagnosis (before, during or after)?

4. What is your current activity level?
    a. How often do you exercise? For how long? What type of exercise do you do?

5. Have you received any form of rehabilitation for cancer, sepsis or frailty?
    a. If yes, can you tell me more about this (what type, for how long)?
        i. Did you find it helpful? Why or why not?
    b. If no, why not?
        i. Would you have like to have rehabilitation services during your cancer treatment? (what type, when)

6. What information or education was available to you (related to rehabilitation) during or after your cancer, sepsis, or frailty diagnosis?
    a. When did this occur?
    b. What did you need more of?

7. What form of education do you think would be most beneficial?
    a. What content would be beneficial?
    b. How would it be the best format to deliver this type of information?
    c. Who should deliver this information?

8. What are your current rehabilitation related needs?

9. What are your current barriers to participating in exercise?

10. Do you have anything else to add about rehabilitation for cancer, sepsis, or frailty?

Semi-Structured Focus Group Guide

Hi everyone! I want to start by welcoming you to this focus group and thanking you for taking the time to participate in our discussion. Today, we will be discussing the rehabilitation potential among people with sepsis, cancer and/or frailty.

Before we begin, we need to disclose our financial support – This project has received financial support from Sepsis Canada and the Canadian Frailty Network. Other than that – we have no conflicts of interest to report.

This is an outline of what we will discuss today. After I give a brief introduction to our processes, I will provide a study overview, some background information and what we know so far from this project. Then we will begin our focus group discussion questions. We will end by discussing next steps.

Some important things to remind you of: Our discussion today will be recorded so we can review and analyze it at a later time. All of your comments and responses to questions will be kept completely confidential. Your name and any other identifiers will be removed from all written records. Any of the materials that come out of our discussion will be evaluated only by staff working on this study and will be kept secure and protected.

The focus group today will last 60 to 90 minutes. I want to say that we acknowledge that technical difficulties may happen! If you get kicked off, then we ask that you come back on as soon as you can, and we will fill you in on what you missed. If we freeze or get kicked off, be patient with us, and we will pick up where we left off.

I will be asking many different questions and I would like as much feedback from you as possible. Your participation and opinions are important and there are no right or wrong answers. We expect that all participants will respect the ideas and opinions of other members; everyone's viewpoint is important, even when they do not align.

A bit of an overview of what led to this project. Two in five Canadian older adults will be diagnosed with cancer. Sepsis is a life-threatening illness where the body overacts to an infection. Cancer survivors have 2 times higher risk of developing sepsis. Further, over 1 million community-dwelling Canadians are frail. The majority with frailty are older adults. These are three common conditions in Canadians – and while frailty, sepsis and cancer have been investigated individually, their interaction is not well understood, although clinical observations note that they often intersect. Additionally, the potential for rehabilitation to improve frailty outcomes for survivors of cancer and sepsis isn't well understood.

The focus group today is part of a larger study. The overall aims of this study as a whole is to explore the potential for rehabilitation amongst the intersection of frailty, sepsis and cancer and create resources to improve survivor experience. Today, we will focus on the last two aims: To determine the components that should be included in educational resources for individuals with sepsis and/or cancer and to determine the optimal format of educational resources being created.

The overall study is broken down into 4 phases: First, we conducted a scoping review on the intersection of cancer, sepsis, and frailty. Within this review we did find that frailty is a common outcome in those with sepsis and cancer in the early post operative period. Next, we conducted interviews to explore patient, caregiver and health professional experiences related to cancer and

sepsis. We'll summarize some of those results on the next few slides. We are currently in phase 3, the focus group. Upon completion, we will move on to phase 4, where we will create educational resources for patients and caregivers and for health professionals on cancer, sepsis and frailty and the potential for rehabilitation to improve survivor outcomes.

Looking at the results from the interview study which we will build on today:
There were four key themes that emerged.

1. First, Rehabilitation received misses the mark. We heard that rehabilitation is valued, but it is provided inconsistently. We also heard that people felt once they were out of hospital, they were on their own.
2. Barriers to rehabilitation exist on multiple levels. We heard that external and internal influences affect availability of rehab services, and that social support affects the ability to participate in rehabilitation
3. A gap exists in the education received. We heard that education for patients and caregivers on cancer and sepsis is delayed which leads to fear and anxiety. The mode of education delivery often lacks personalization and understanding. We heard participants had to search for information on sepsis themselves. We also heard from health care professionals that there is a lack of information on these conditions in entry-level health education.
4. Educational needs exist related to cancer, sepsis, frailty and rehabilitation. We heard that more information is needed on the link between cancer and sepsis. There is a need for flexibility in the mode of education delivery. We heard that the provider of education directly influences the impact of the information received. For example, most want to hear from the 'expert' in the area, but patients really value the advice given from there physician over all others. We also heard that content needs to be specific and meaningful.

So we have learned a lot so far, but now we would like to hear more of what you think regarding educational resources. During this time, I would like to have you discuss your perspective on the use of education modules for individuals with cancer and sepsis and discuss the content to include and mode of content delivery.

What are your thoughts on the need for educational materials for individuals (patients, caregivers, health professionals) involved with or affected by sepsis, and/or cancer?

What content do you think should be included in these educational modules?

- Related to cancer and sepsis.
- Related to frailty outcomes.

This is the same question: What content do you think should be included in these educational modules? Here, we would like you to consider content from a perspective of rehabilitation for these conditions.

What are your thoughts on the mode of delivery (i.e., ways to deliver the educational material)?

- Perceived strengths/weakness, pros/cons of different modes
- Consider setting
- Consider the viewer or user

What else do you think we should consider when creating modules related to cancer, sepsis, and frailty?

Have we missed asking a question that you believe is important?

Do you have anything else to add?

Thank you everyone!
